# Born in Brazil: shining a light for change

**DOI:** 10.1186/s12978-016-0247-4

**Published:** 2016-10-17

**Authors:** Maria Regina Torloni, Ana Pilar Betrán, José M. Belizán

**Affiliations:** 1 Department of Internal Medicine, Evidence Based Healthcare Post-Graduate Programme, São Paulo Federal University, São Paulo, Brazil; 2 Department of Reproductive Health and Research, UNDP, UNFPA, UNICEF, WHO, World Bank Special Programme of Research, Development and Research Training in Human Reproduction, World Health Organization, Geneva, Switzerland; 3 Institute for Clinical Effectiveness and Health Policy (IECS), Buenos Aires, Argentina

## Abstract

The *Birth in Brazil* study is the largest national hospital-based survey in Brazil regarding birth practices. Conducted in 2011–2012, it collected information from 266 public and private healthcare facilities and interviewed nearly 24,000 postpartum women. It is also the latest effort to map out how labor and delivery are managed in this county in the 21st century. The journal Reproductive Health has published a supplement including 10 articles presenting the results of a series of analyses using this valuable resource.

These articles describe a range of practices, determinants and risk factors that affect women and their babies in Brazil, a country of paradoxes. In the era of overmedicalization and high-tech medicine – arguably –, these articles highlight the unprecedented rates of cesarean sections in Brazil and differences between the public and the private sectors. It provides evidence for the need for adequate human resources, medications and emergency care equipment in many settings; and explains the use of non-evidence based interventions during labor and delivery. On the other hand, these studies also point to promising interventions that could be used to change this situation not only in Brazil but also in other countries facing similar challenges.

Brazil, one of the largest country in the world in terms of territory and population, has many socioeconomic contrasts and paradoxes. There are large disparities in the obstetric care received by women in the highest socioeconomic strata, mainly managed by private health insurance, compared to those in the lowest strata who are fully dependent on the often deficient and overburdened free public health system. In this Supplement, *Reproductive Health* publishes a series of articles that describe what is happening in Brazil in relation to birth practices, their consequences and potential ideas for interventions that could help to change this picture.

The studies in this issue used data from the largest national hospital-based survey (the “*Birth in Brazil*” study) which collected information from 266 public and private healthcare facilities and interviewed nearly 24,000 postpartum women between 2011–2012 [1]. This landmark study, coordinated by Maria do Carmo Leal from Osvaldo Cruz Foundation (Fiocruz) in collaboration with several prestigious Brazilian scientific institutions, mapped out and synthesized for the first time the situation about birth practices in the county in the 21^st^ century. The many highly informative publications from this laborious and carefully conducted study provide a revealing picture of how labor and delivery are currently managed in the country and offer insightful data on the determinants, magnitude and consequences of the use of interventions during labor and delivery in Brazil. More importantly, these studies also point to possible ideas, actions and interventions to improve obstetric care in the country.

One of the most striking characteristics of Brazil is the high rates of cesarean deliveries (CD), especially in the private sector, where 80–90 % of all women are delivered by this route [2, 3]. Despite the lack of evidence indicating substantial health benefits of CD rates beyond a certain threshold, and growing evidence that CD may be associated with poorer maternal, neonatal, childhood and long term outcomes [4–9], the use of this surgery has steadily increased over the last thirty years in most high and middle-income countries [10]. According to official national estimates, Brazilian CD rates rose from 38 % in 1994 to 50 % in 2009 and reached 57 % in 2015 [3, 10]. This means that each year, nearly 1,700,000 caesareans are performed in Brazil, many of which are probably unnecessary from a medical perspective [11, 12].

Indeed, the probability of having a CD in Brazil is heavily influenced by non-medical factors. Brazilian women who have more education, are in their first pregnancy and who receive prenatal care in the private sector have a significantly higher probability of delivering by cesarean section (C-section) than those without these characteristics [11, 13–16]. The study by Gama and Nakamura-Pereira et al. [17] reports that while 52 % of all women interviewed in the national survey delivered by cesarean, CD rates were 88 % in the private hospitals compared to 43 % in the public hospitals. Moreover, as reported in a previous publication using the same dataset, 80 % of the C-sections in the private sector were electively scheduled and performed before the onset of labor [18]. Gama and Nakamura-Pereira et al. also analyzed the data using the Robson classification and report that nulliparous women in spontaneous labor at term, with a singleton cephalic fetus (Robson group 1) delivered in private hospitals had more than twice the rate of CD than in public hospitals (44 % versus 18 %, respectively) [17]. These results suggest that clinical practice varies enormously in different settings in Brazil and may be heavily influenced by societal and other non-medical factors. Although the rates of CD in nulliparas are much lower in the public than in the private sector, they are still considerably higher than what would be considered medically reasonable. However, as Dias et al. [19] report, this could be improved with the adoption of simple interventions. In their analysis of nulliparous women delivering in public hospitals, these authors concluded that information and support for vaginal birth during antenatal care, avoiding early admission (before 4 cm dilation), and promoting the use of good clinical practices during labor could reduce unnecessary CD in nulliparous women with a singleton cephalic fetus.

C-sections are perceived by some women as safer than vaginal deliveries [20–24]. This misconception may be a potential contributor to the high rates of scheduled C-sections in Brazil, especially maternal-request cesareans performed in the private sector. However, as previously reported in other studies, and contrary to popular belief, elective C-sections are not without risk [8, 25–29]. In one of the articles of this issue, Domingues et al. show that women with an elective C-section had over twice the risk of maternal near miss, after adjustment for pregnancy complications, social and demographic variables, and antenatal care [30]. The increased risk for maternal near miss in women submitted to elective C-sections in Brazil is especially worrisome when we look at the findings of Bittencourt et al. that show the low quality of hospital services available for pregnant women [31]. According to these authors, less than 35 % of the maternity wards across the country have adequate human resources, medications and emergency care equipment to ensure survival and adult intensive care beds. These findings underscore the need to avoid unnecessary CD in Brazil, especially in settings that lack the facilities and/or capacity to properly conduct safe surgery and treat surgical complications, as recommended by the 2015 WHO Statement on Caesarean Section Rates [9]. In addition, the *Birth in Brazil* study reported that the rate of preterm births (PTB) in the 2011–2012 survey was 11.5 % [32] which is nearly twice that of European countries [33, 34]. Leal et al. conclude that the high rate of PTB in Brazil is due to a high proportion of provider- initiated late preterm births, especially among women being delivered in private healthcare facilities and those with a previous CD [32]. This suggests that many cases of late PTB in Brazil may be caused by iatrogenic prematurity in women scheduled for elective CD, with incorrect gestational age assessment. The practice of delivering late preterm infants is associated with increased morbidity, including the need for resuscitation in the delivery room, as pointed out by the study by Moreira et al. [35].

The aim of care during normal labor is to achieve a healthy mother and child with the least possible level of intervention that is compatible with safety. Since women and their babies can be harmed by unnecessary practices, there should be a valid reason to interfere with the natural birth process [36]. In Brazil, the use of non-evidence based interventions during labor and delivery (e.g. routine episiotomy or oxytocin drips) is still high, while the adoption of beneficial, evidence-based practices (i.e. partographs) is still low. In fact, best practices during labor were adopted in less than half of all women delivered in the *Birth in Brazil* study [37]. However, da Gamma et al. point to a solution for this situation: include more nurse-midwives in the healthcare teams. These authors report that in Brazilian settings where a nurse-midwife was in charge of conducting labor and delivery, the use of good practices recommended by WHO was significantly more frequent, and the use of obstetric interventions was significantly less frequent, than in settings where labor and delivery were conducted exclusively by physicians [38]. Moreover, the study also concluded that the presence of a nurse-midwife in the maternity care team reduced the rate of CD, a finding confirmed by another Brazilian study involving private hospitals [39]. In addition, basic human and social skills are also important for health professionals caring for women in labor. In fact, good communication and a respectful, supporting and empathic relationship between the healthcare providers and laboring women are part of the good clinical practices promoted by WHO ever since 1996 [36]. Baldisserotto et al. reported that this type of communication, along with the presence of a birth companion throughout labor and delivery, were associated with increased satisfaction of Brazilian women with the care received during childbirth [40]. A recent WHO statement emphasizes the importance of providing respectful care for laboring women [41].

Being born vaginally increases the success of breastfeeding practices, an essential aspect of maternal and infant health. The benefits of breastfeeding during the initial years of life cannot be overstated. Early suckling stimulates breast milk production and facilitates the release of oxytocin, which helps the uterus contract reducing postpartum blood loss. The colostrum contained in the first breast milk is highly nutritious and has antibodies that protect the newborn. Early initiation of breastfeeding also fosters bonding between mother and child. In the *Birth in Brazil* study, babies born vaginally had almost three times the chance of being breastfed in the first hour after birth than those born by C-section [42].

Paradoxically, the urge of Brazilians to plan and control how and when to deliver, that can be inferred from the high elective CS rates in the national survey, does not seem to run in parallel with the need to plan if and when they want to get pregnant. According to Theme et al., less than 45 % of the post-partum women interviewed in the national survey reported that their pregnancy had been intended [43]. Worldwide, it is estimated that approximately 40 % of all pregnancies are unintended, ranging from 35 % in Africa to 56 % in Latin America and the Caribbean region [44]. The findings of Theme et al. point to the need to implement strategies to overcome barriers and increase the access to high quality of family planning services in Brazil.

The series of studies in this issue *Reproductive Health* point to the paradox of obstetric care in Brazil and its consequences, in other words: overmedicalization of childbirth, alongside with substandard quality of care, leading to potentially avoidable adverse maternal and neonatal outcomes. This situation can change if policy makers, stakeholders, physicians, nurses, midwives and women in Brazil join forces and take action. The findings of the studies reported in this *Reproductive Health* issue also serve as a warning to what may (or will soon) be happening in other middle-income countries, if nothing is done to halt and reverse the increasing CD trend. On the other hand, these studies also point to promising, and often simple and low-cost, interventions that could be used to change this situation in Brazil and in other countries facing similar challenges.

Kant said: “Dare to know”. This is what the *Birth in Brazil* study set out to achieve by taking a closer and evidence-based look at what it is happening in their country. The aim of the studies published in this *Reproductive Health* supplement, as well as the aim of this editorial, is not to criticize health authorities, healthcare providers or women but to raise awareness about a system that does not always seem to have the ‘best care for the woman and baby’ as its primary goal. Knowledge is a double edge sword; it saves us but also burdens us with the responsibility for action. More research is now needed to move beyond describing problems and barriers, to identify and test solutions. However, work will be slow and intense since changes, especially sustainable changes, do not occur overnight. Long-held societal and cultural routines, practices, views and beliefs are never easy to put aside, but we are confident that Brazil is reaching a critical mass of awareness, knowledge and will to improve obstetric care.
